# Development and Implementation of a Stress Monitoring Paradigm Using Virtual Reality Simulation During the COVID-19 Pandemic

**DOI:** 10.7759/cureus.53450

**Published:** 2024-02-02

**Authors:** Andrei Torres, Binh Nguyen, Bill Kapralos, Sridhar Krishnan, Douglas M Campbell, Lindsay Beavers, Adam Dubrowski, Venkat Bhat

**Affiliations:** 1 maxSIMhealth Group, Ontario Tech University, Oshawa, CAN; 2 Electrical, Computer, and Biomedical Engineering, Toronto Metropolitan University, Toronto, CAN; 3 Allan Waters Family Simulation Program, Unity Health Toronto, Toronto, CAN; 4 Neonatal Intensive Care Unit, St. Michael's Hospital, Toronto, CAN; 5 Li Ka Shing Knowledge Institute, St. Michael's Hospital, Toronto, CAN; 6 Pediatrics, University of Toronto, Toronto, CAN; 7 Physical Therapy, University of Toronto, Toronto, CAN; 8 Psychiatry and Interventional Psychiatry, St. Michael's Hospital, Toronto, CAN

**Keywords:** virtual reality (vr), stress experiences, covid19, virtual reality simulation, moral distress, digital intervention, healthcare simulation

## Abstract

Healthcare providers, particularly during the COVID-19 crisis, have been forced to make difficult decisions and have reported acting in ways that are contrary to their moral values, integrity, and professional commitments, given the constraints in their work environments. Those actions and decisions may lead to healthcare providers' moral suffering and distress. This work outlines the development of the Moral Distress Virtual Reality Simulator (Moral Distress VRS) to research stress and moral distress among healthcare workers during the COVID-19 pandemic. The Moral Distress VRS was developed based on the agile methodology framework, with three simultaneous development streams. It followed a two-week sprint cycle, ending with meetings with stakeholders and subject matter experts, whereby the project requirements, scope, and features were revised, and feedback was provided on the prototypes until reaching the final prototype that was deployed for in-person study sessions. The final prototype had two user interfaces (UIs), one for the participant and one for the researcher, with voice narration and customizable character models wearing medical personal protective equipment, and followed a tree-based dialogue scenario, outputting a video recording of the session. The virtual environment replicated an ICU nursing station and a fully equipped patient room. We present the development process that guided this project, how different teams worked together and in parallel, and detail the decisions and outcomes in creating each major component within a limited deadline. Finally, we list the most significant challenges and difficulties faced and recommendations on how to solve them.

## Introduction

Since COVID-19 was declared a pandemic, governments worldwide have issued lockdowns/stay-at-home orders, leading to the shutdown of businesses and educational institutions, the scaling back and postponement of non-emergency medical care, and fundamental changes to healthcare provision [1]. However, the need for rapid and changing contingency planning, shortages in personal protective equipment, resource shortages, and the inability to provide the ethical caring called for by professional codes of ethics placed healthcare providers in what has been described as a “war zone” [2]. Healthcare providers have been forced to make difficult decisions and have reported acting in ways that are contrary to their moral values, integrity, and professional commitments, given the constraints in their work environments [3-4]. Moral suffering can ensue when a healthcare provider’s moral foundation is threatened or violated by witnessing or participating in decisions or actions that degrade their integrity [5]. In addition, moral distress may arise when healthcare providers cannot translate their moral choices into action because of internal or external constraints [6]. As these events and other workplace stressors accumulate, healthcare providers’ capacities to provide the level of care they desire can be diminished.

Given the potential impact of stress and moral distress (henceforth called S&MD) on healthcare workers and healthcare in general, an interdisciplinary team was formed to examine S&MD with the application of digital technologies, including virtual reality. The team aimed to determine the feasibility of creating a digital environment and implementing digital interventions to better understand the phenomenon of S&MD by developing platforms to examine moral distress and monitor participants over time to develop a better understanding of the continuum of S&MD. One of the platforms was the Moral Distress Virtual Reality Simulator (Moral Distress VRS), developed to research S&MD amongst healthcare workers during the COVID-19 pandemic.

The goal of the Moral Distress VRS was not to explore interaction methods but to provide a more immersive experience to participants by virtually inserting them in a mid-pandemic ICU-like environment, and monitor their responses in a potentially stressful situation. Marín-Morales et al. [7] present a systematic review of how head-mounted VR equipment is being used in emotion research, and Han et al. [8] assert that immersive VR tends to evoke strong reactions to stressful or dreadful situations.

## Technical report

In this technical report, we outline the development flow and technical details of the Moral Distress VRS, developed under a six-month deadline (from January 2021 to May 2021), exploring its focus, goals, the equipment and setting used, the development approach, and its final output, which was used to conduct a series of human-based experiments from May 2021 to August 2021 at St. Michael's Hospital in Toronto, Canada. The research protocol has been described by Nguyen et al. [9] and study results have been presented by Espinola et al. [10].

Hardware

Due to the limited deadline, time had to be carefully and properly managed. Therefore we decided to adopt a consumer-grade VR head-mounted display (HMD or headset) that was readily available for purchase and selected the Meta Quest 2 [11] (at the time called Oculus Quest 2) VR headset (Oculus Studios, Menlo Park, California, United States).

Although it is a self-contained device, the Meta Quest 2 can also be used connected to a computer in a mode called Link. This mode allows the Meta Quest 2 to be used as a computer-based VR headset, meaning the computer must perform all the processing to run the applications. We adopted the Link mode because it provided us with greater control regarding privacy since it bypasses the necessity of using a Meta/Facebook account. Furthermore, all devices were used in offline mode.

As the computer that executed the Moral Distress VRS and controlled the Meta Quest 2, we adopted a 15.6" laptop with the following specifications: Intel Core i7 10750H 2.6 GHz CPU, 16 GB RAM, 512 GB SSD, 1920 x 1080 (Full HD) screen, GeForce RTX 2070 SUPER Max-Q 8GB GPU.

Design

The development process of the Moral Distress VRS was based on the agile methodology framework. As defined by Atlassian Corporation, Sydney, Australia, [12], agile is a group of methodologies that demonstrate a commitment to tight feedback cycles, continuous improvement, and frequent increments that let the team gather feedback on each change and integrate it into future releases at a minimal cost. 

Three development streams occurred simultaneously: i) Script Stream (responsible for creating the dialogue script for the simulation), ii) VR Stream (responsible for creating the VR simulation), and iii) Biopac Stream (responsible for the physiological data collection); this paper focuses on the VR Stream.

Each development stream was organized as follows: i) Script Stream: one lead writer, two support writers; ii) VR Stream: one lead developer; iii) Biopac Stream: one lead developer. The three development streams were supported by a group of six subject-matter experts (SMEs) in the fields of psychiatry, VR, biomedical engineering, and simulation.

**Figure 1:**
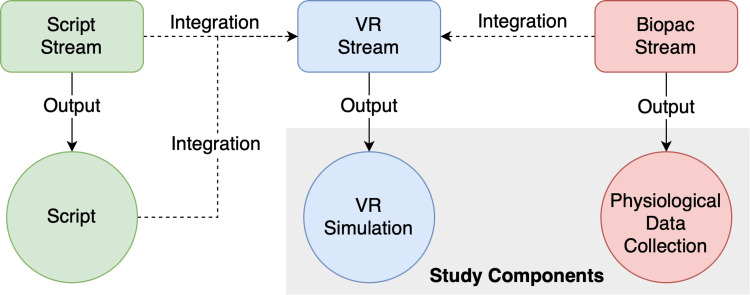
Graph showing the outputs and connections of the three development streams.

Although the three streams could be developed mostly independently, there was still some codependency between them (Figure 1): the VR Stream used the Script Stream output to determine which features should be researched and implemented (and provided feedback on feasibility). In parallel, the VR Stream and Biopac Stream worked together to determine if communication between the equipment was possible (and how much they could be integrated).

**Figure 2:**
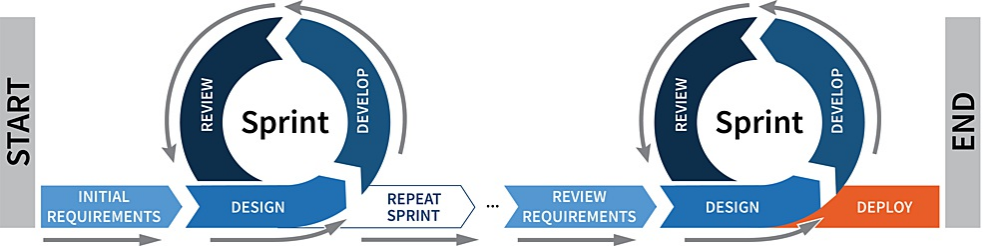
Project development cycles.

All streams followed a two-week sprint cycle, ending with meetings with stakeholders and SMEs, whereby the project requirements, scope, and features were revised, and feedback was provided on the current prototypes (Figure 2) until reaching the final prototype that was deployed for the in-person study sessions at the end of May. In total, from January to May, ten sprint cycles were held by all three development streams.

The VR Stream development cycles focused on the following features, which were developed in parallel: i) environment, ii) characters, iii) user interface (UI) and user experience (UX), iv) data collection, v) Biopac Stream integration, and vi) Script Stream integration.

Environment

The environment (the location where the VR simulation takes place) was defined by the Script Stream. Placeholder environments were used during the development of the initial version of the script. Reference gathering (photos and three-dimensional (3D) assets) started once it was determined that the environment would be an ICU nursing station, with a patient room containing equipment related to a COVID-19 case (a patient in critical condition connected to a ventilator). Next, those references were discussed with the SMEs, which explained what key elements had to be present and how to correctly present them (e.g., the layout of the ICU nursing station).

**Figure 3:**
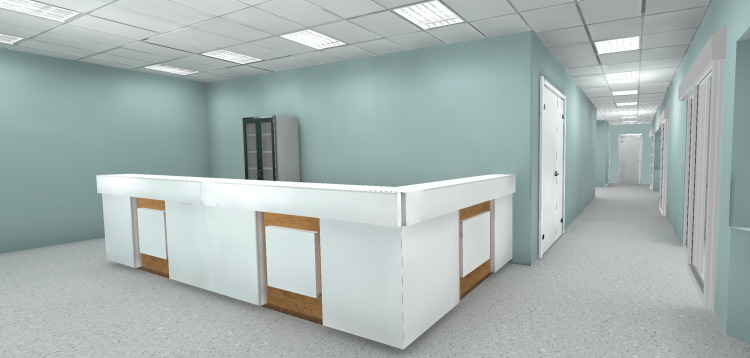
Nursing station and hallway.

**Figure 4:**
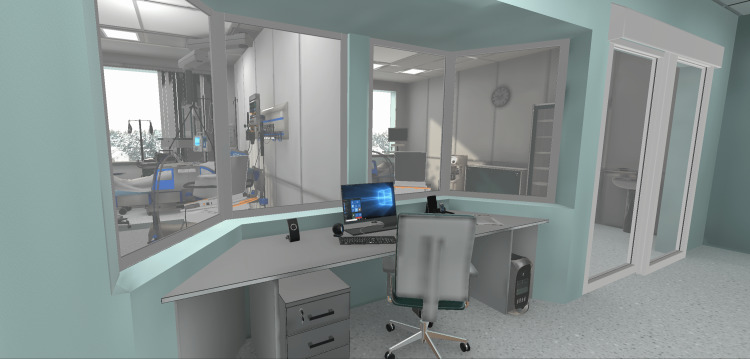
Patient rooms monitoring area.

**Figure 5:**
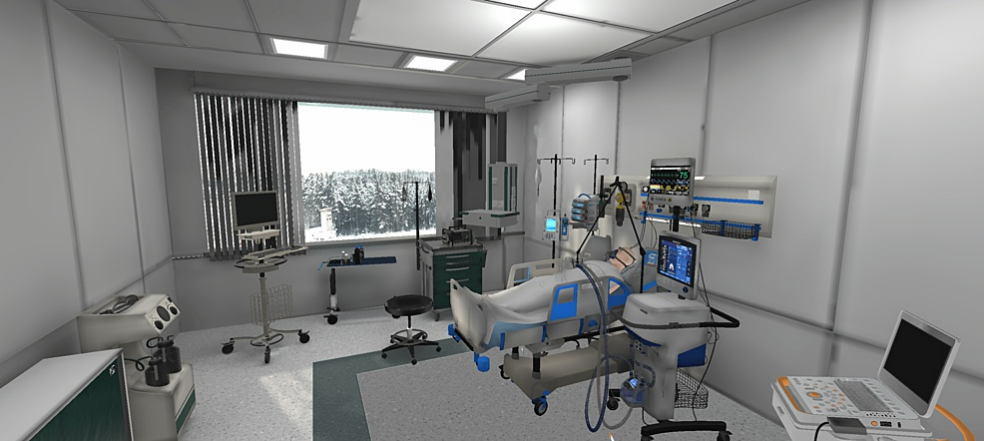
Fully equipped patient room.

Based on those discussions, the search for 3D assets to compose the environments and the development of the actual virtual environments began. We adopted Unity (Unity Software, Inc., San Francisco, California, United States), a cross-platform game engine, to develop the Moral Distress VRS due to the prior expertise of the members involved, and also due to the availability of a library that allowed direct communication between Unity and the physiological data collection system adopted (Biopac). At the time, such a library was not available for other game engines (e.g., Unreal (Epic Games, Inc., Cary, North Carolina, United States) or Godot (Godot Foundation, Uitgeest, Netherlands). Subsequently, we purchased environment assets from the Unity Asset Store (Unity Software, Inc., San Francisco, California, United States) [13], TurboSquid (New Orleans, Louisiana, United States) [14], and CG Trader (Vilnius, Lithuania) [15] and combined them to create the environments that would fit the project requirements (Figures 3, 4, 5).

Characters

As with the environment, the Script Stream also defined the characters (avatars). Nevertheless, the development of this feature started by researching how to implement the characters. The following options were defined: i) create from scratch, ii) purchase prepackaged ready-to-use characters, and iii) modify existing assets to address the project requirements. Due to time and resource constraints, option i) was not feasible. Furthermore, in January 2021, the availability of characters wearing medical personal protective equipment (PPE) on the Unity Asset Store was severely lacking, therefore eliminating option ii). Thus, it was decided to go with option iii).

We adopted the Unity Multipurpose Avatar (UMA) system [16], a free and open-source character creation and modification system. The developer must use a base model provided by UMA, acquire one through a third party, or import a model prepared to be used by UMA. The level of customization depends on how the 3D assets were implemented in UMA (e.g., to customize the character weight, blend shapes must have been defined in the original 3D model of the character).

Unfortunately, as stated previously, PPE assets were not readily available at the Unity Asset Store (either for UMA characters or other types of characters), which led us to look elsewhere. Finally, we found Daz 3D (Daz Productions, Inc., Salt Lake City, Utah, United States), a company with a 3D marketplace containing over five million inter-compatible assets for Daz Studio (their 3D software), which could be exported to other 3D applications [17]. The Daz 3D marketplace had PPE assets available, which the VR Stream team presented to the SMEs to be assessed. Once the SMEs selected and approved the assets, the VR Stream team began porting them to the UMA system.

**Figure 6:**
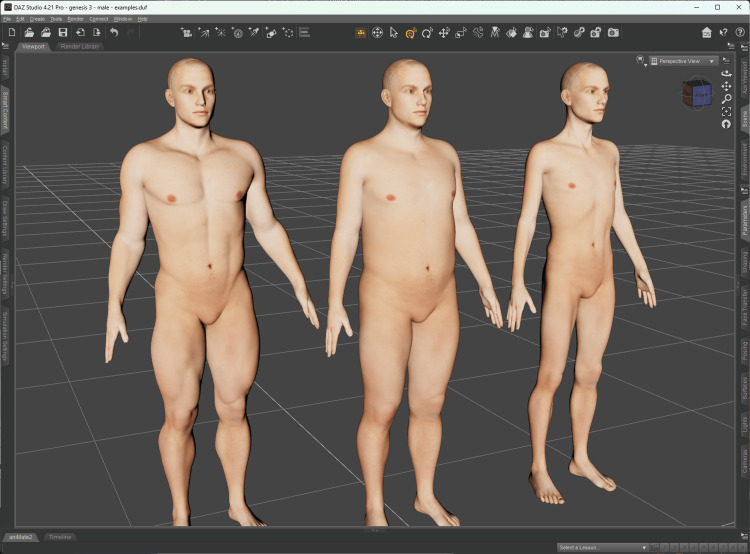
Daz Studio* showing the Genesis 3 male model with different body settings. *Daz Productions, Inc., Salt Lake City, Utah, United States

**Figure 7:**
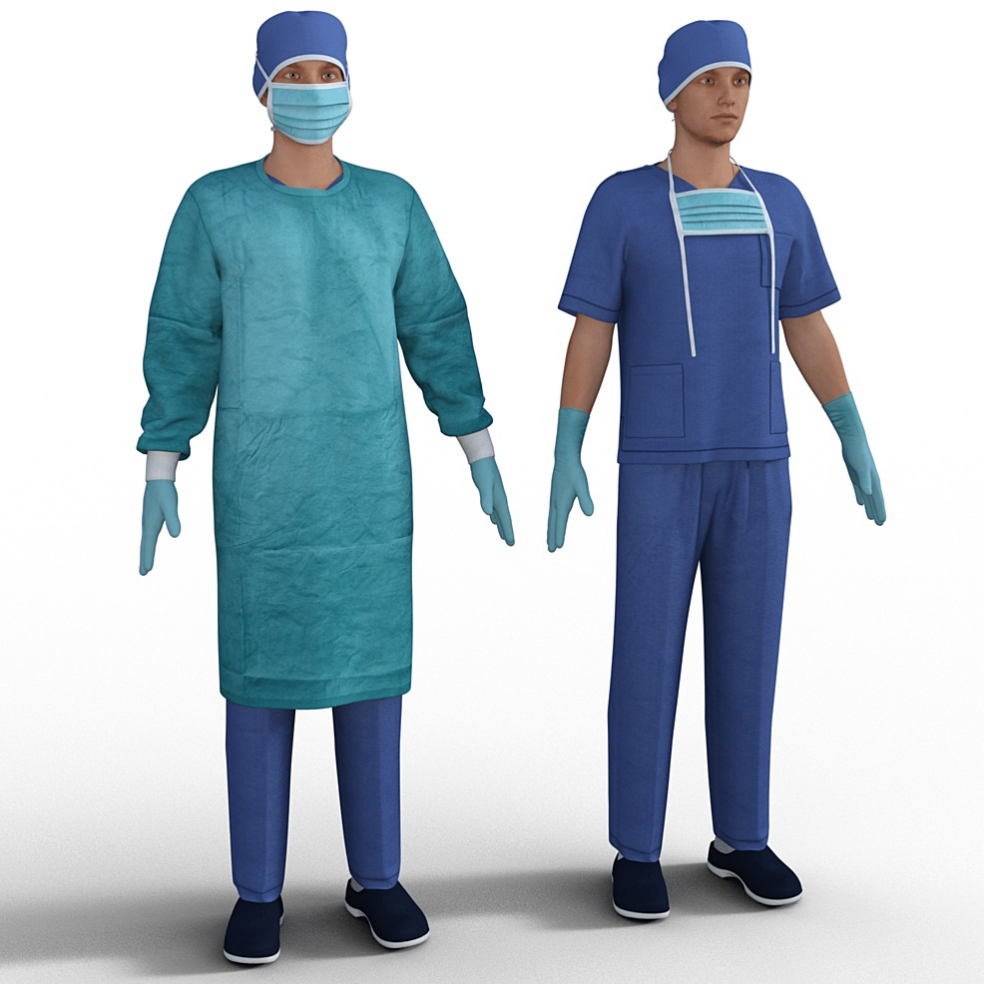
Two usage examples of the H&C Medical Scrubs Set*. *Daz Productions, Inc., Salt Lake City, Utah, United States

**Figure 8:**
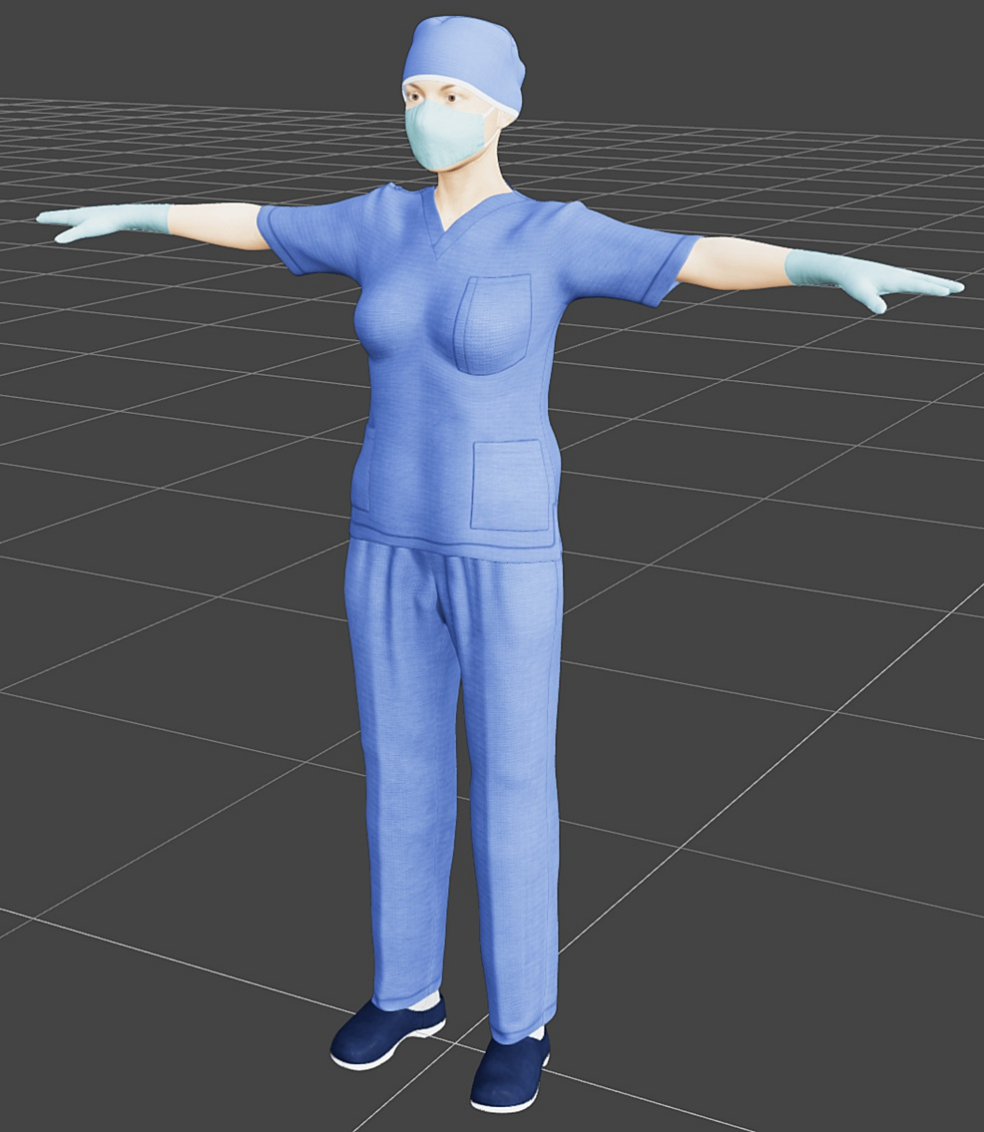
Genesis 3 female model* with H&C Medical Scrubs Set*. *Daz Productions, Inc., Salt Lake City, Utah, United States

As a starting point, we used the Daz Studio's Genesis 3 [18] model set to create our own UMA base model, which provided us with models containing "blend" shapes to adjust weight and muscle (Figure 6). On top of the Genesis 3 model, we applied the Daz Studio's H&C Medical Scrubs Set for Genesis 3 Male(s) package [19] (Figure 7), which contained assets such as a mask, gloves, scrubs, headwear, and surgery gown. Although the H&C Medical Scrubs Set was created for the male version of the Genesis 3 model, we adapted it to fit the female version (Figure 8).

**Figure 9:**
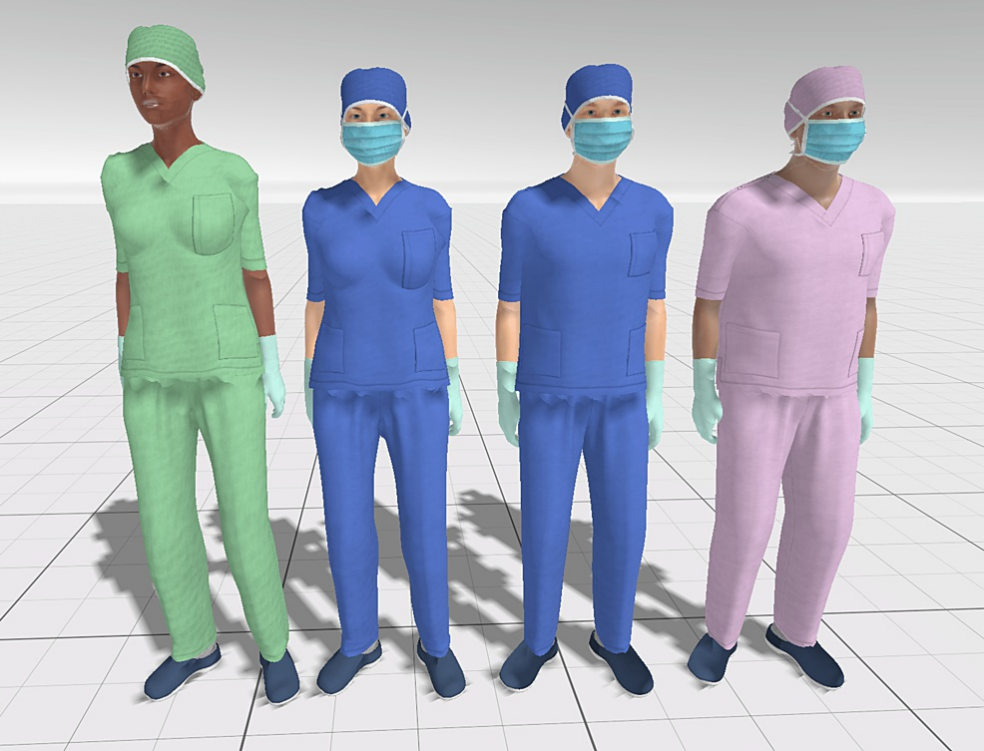
Example of character customization. Four characters with different body types, heights, and skin tones wearing scrubs of various colors (green, blue, and pink).

The next step was to generate the UMA models. To that end, it was first necessary to export from Daz Studio into Blender (Blender Foundation, Amsterdam, Netherlands), a free and open-source 3D creation suite. Youtuber Secret Anorak created a detailed guide explaining the whole exporting procedure from Daz Studio, adjusting in Blender, importing in Unity, and creating a UMA base model [20]. We created two UMA base models, with customizable skin tone, eye color, height, and weight, both with a complete set of PPE clothes with customizable colors (Figure 9).

UI and UX

**Figure 10:**
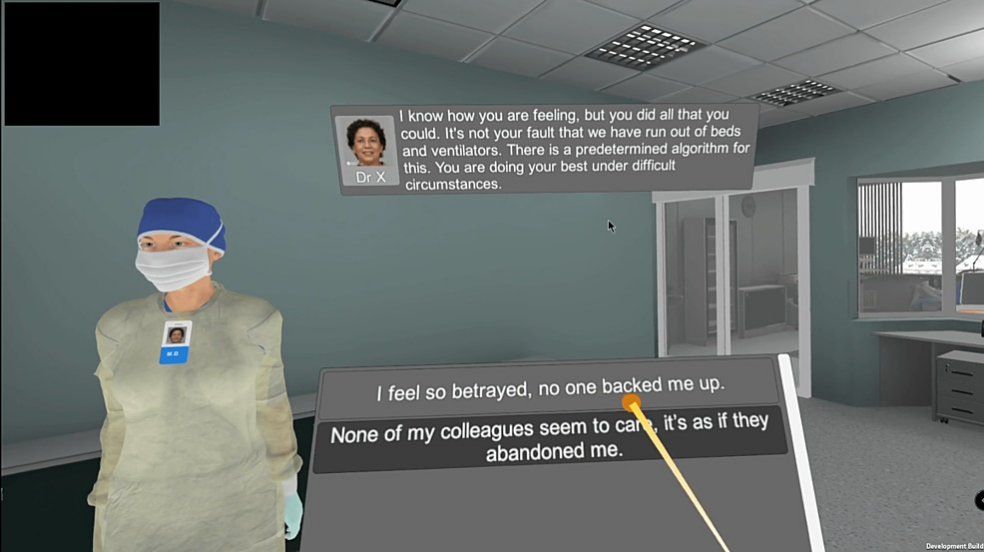
Player user interface (UI) showing non-playable character (NPC) information, dialogue text overhead, and list of choices below.

**Figure 11:**
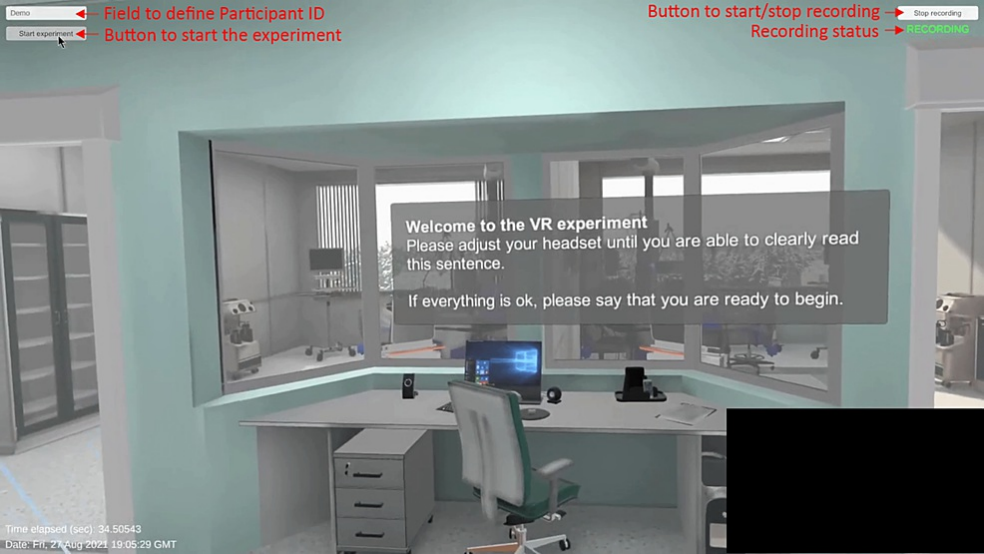
Researcher user interface (UI) that showed the participant's point of view and allowed the researcher to control the session.

Two UIs were developed: i) a player UI (Figure 10) and ii) a researcher UI. The player UI was visible to the participant while wearing the VR headset, allowing interaction with the scenario. The researcher UI (Figure 11) was visible only to the researcher, providing control of the session (defining participant ID, starting/stopping recording, and starting/stopping the session). Starting the Moral Distress VRS scenario starts both the player and researcher UIs simultaneously, outputting the researcher UI to the primary monitor of the computer and the player UI to the VR headset.

The player UI was one of the main areas of development since it defined how the participant would interact with the virtual environment. The Dialogue System for Unity package (Pixel Crushers, Mississauga, Ontario, Canada) [21] was used as the base engine because it provided a conversation system with complete dialogue UI elements and interactions, VR support, audio playback, and a visual node-based editor with support for conditions and triggers (more information in the Script Integration section). Moreover, since all non-playable characters (NPCs) were wearing masks due to the ICU environment and the COVID-19 pandemic, to help the participant better identify the speakers, badges that included their fictional names and photographs were attached to all NPC models and the same information was visible in the dialogue text in the overhead portion of the player UI (Figure 10).

Regarding input methods, although the Meta Quest 2 included hand-tracking capabilities in January 2020, during test sessions, we found it lacked reliability and precision compared to the Meta Quest 2 controllers. Additionally, the only interaction necessary from the participant was to point toward the desired choice and click a button to confirm. To further facilitate that, all buttons of both controllers were mapped such that they performed the same action (click the highlighted button in the UI), and we added a ray coming out of both virtual hands’ index fingers to visually represent where the participant was pointing and what they were interacting with (Figure 10). The idle state of the ray is blue, and it turns yellow when the ray intersects a UI element, with an orange sphere in the area where it touches the UI.

Moreover, electrocardiogram, electrodermal activity, photoplethysmography, and respiration impedance sensors were attached to the participant's torso and one hand, to monitor their primary heart rate, respiratory rate, oxygen saturation, and skin conductance. To prevent any negative effects on the data collection, we decided to ask the participant to indicate the dominant hand, which would be used to interact with the scenario, and the off-hand would have the sensors attached. Regardless, both hands had to hold the controllers to correctly position the participant’s virtual avatar hands in the virtual environment.

The scenario focused on having the participants experience a stressful situation that required them to follow a decision that went against their morals. In this specific scenario, the participant played the role of a physician working in an ICU during the COVID-19 pandemic, at capacity, having to decide to remove the respirator from an elderly patient with co-morbidities and move it to another patient with a higher chance of survival (following the regional health authority algorithm). Additionally, the participant had to communicate that decision to the patient's partner. As such, the focus was on the dialogue rather than exploring the scenario. In addition, allowing the participant to walk around exploring the scenario could serve as a distraction. Moreover, it was expected that most participants lacked prior experience with VR headsets. Thus, it was decided to have the participant remain seated without the capability of physically walking around, avoiding the need for a room with enough space to allow the participant to move while wearing the VR headset and prevent any possible accidents (e.g., tripping on the cable that connects the VR headset to the computer).

During test sessions with the development team, we experimented with having the player UI stationary and floating around following the player’s point of view. However, we found that having the UI floating around was distracting and interfered with the participant’s field of view if they wanted to look around and inspect the environment.

**Figure 12:**
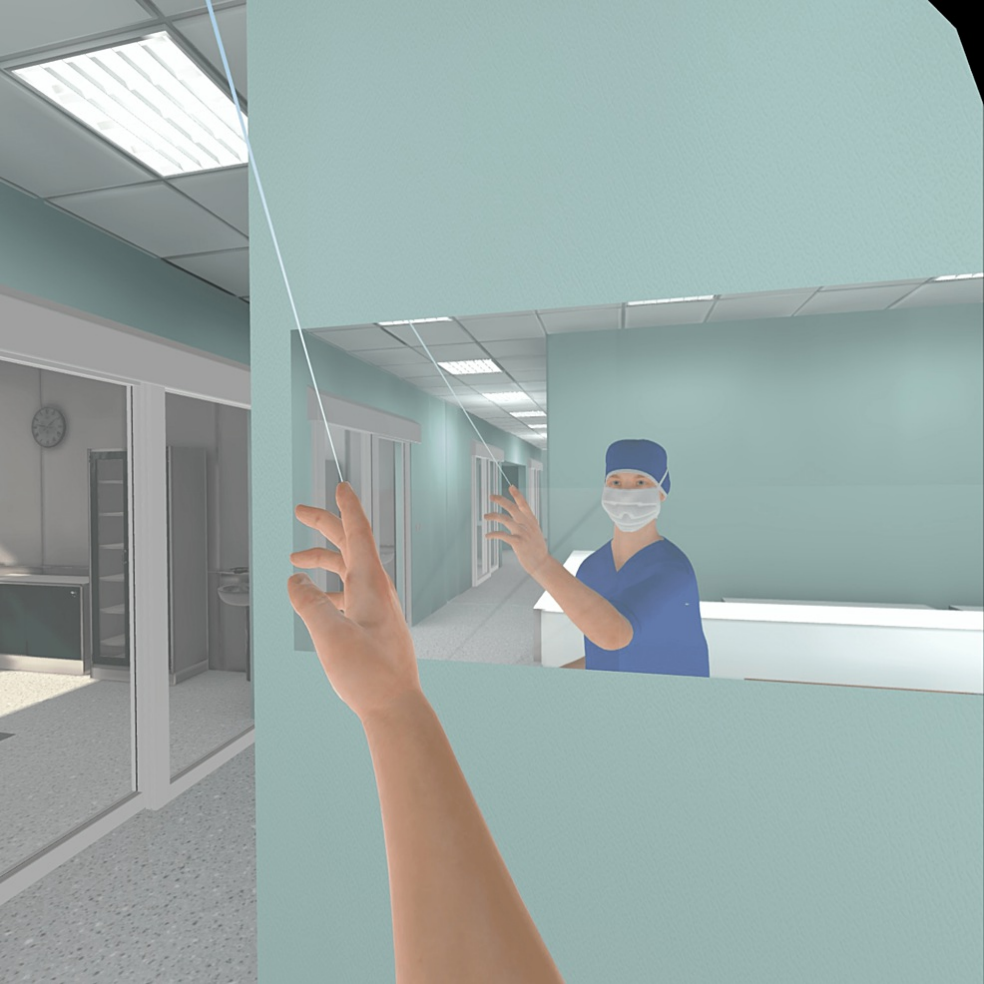
Participant seeing their own virtual body in a mirror inside the VR environment. VR: virtual reality

Since we did not make any assumptions regarding the participants’ prior VR experience, to prepare and guide the participants on how to interact with the VR environment, the scenario started with an interactive tutorial explaining how to use the controllers, look around the room, interact with the UI, and a brief explanation with background information on the case. Furthermore, to provide a better sense of body ownership to the participants, meaning that they would feel as if the virtual body was their own, we included a real-time mirror to reflect the participants’ movement during the tutorial (Figure 12) [22].

During the Moral Distress VRS scenario's development and test phases, we used the Google Cloud TTS (Text-To-Speech) (Google LLC, Mountain View, California, United States) system to generate the voice narration for the NPCs. However, the feedback provided during the test phases was that the TTS audio was too robotic and lacked the emotion needed to convey the gravity needed for the situation. Thus, we switched to voice narration recorded by the project team members and by SMEs in podcast recording.

Lastly, to help further increase the sense of immersion, equipment sounds were obtained from royalty-free libraries (e.g., Freesound, Music Technology Group, Barcelona, Spain) and added to the VR environment, including the sounds of the ventilator machine and the ECG/EKG monitor in the patient room. The ECG/EKG monitor had two states: i) regular heartbeat and ii) cardiac arrest, played according to the participant’s position in the script. Additionally, the sounds were spatially positioned according to their sources, allowing the participants to identify where they were originating from.

Data collection

The Moral Distress VRS scenario collected data in two ways: i) passive and ii) active. The passive data collection was accomplished by recording the participant’s point of view in the virtual environment as soon as the researcher defined the participant ID for the session and started the scenario (after the participant confirmed being ready). To record the participant's point of view, a virtual camera was attached to the 3D object representing the participant’s head, recording a close approximation of the point of view (and not a stereoscopic video representing each eye), and outputting an MP4 video file.

**Figure 13:**
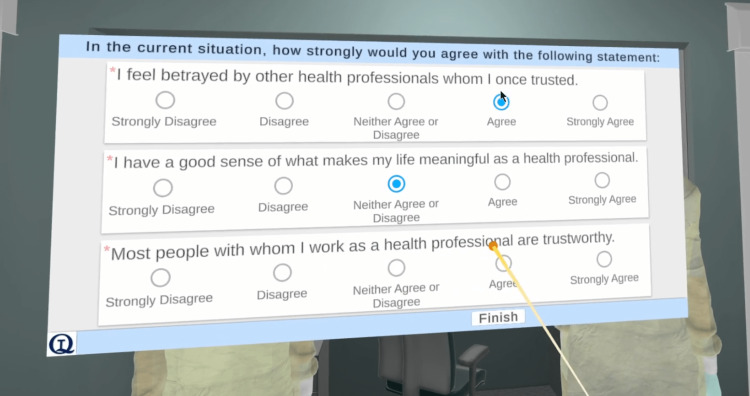
A questionnaire within the VR environment showing three questions with linear scale choices. VR: virtual reality

The active data collection was accomplished by having the participant complete two questionnaires while wearing the headset during the VR session: the Adapted Moral Injury Symptom Scale: Healthcare Professionals Version (MISS-HP) and the Igroup Presence Questionnaire (IPQ). Those questionnaires, validated by prior research [23-24] and presented and explained [9], were implemented using the QuestionnaireToolkit asset [25], available on the Unity Asset Store. The QuestionnaireToolkit provides full VR support (Figure 13), eight types of customizable questions (e.g., slider, linear scale, multiple choice), and saves the responses as a CSV (Comma-Separated-Values) file, which can be easily imported by other software, such as Microsoft Excel (Microsoft Corporation, Redmond, Washington, United States) or IBM SPSS (IBM Corp., Armonk, New York, United States), for data analysis. The IPQ data statistics (mean, standard deviation, median, IQR, and variance) were analyzed to identify areas of improvement (e.g., low realism) and correlated with the qualitative data collected during the experiment sessions [10].

Biopac integration

The Biopac equipment is controlled by the AcqKnowledge [26] software, which handles data acquisition and analysis. Biopac also provides a utility called Unity Interface [27] that allows bidirectional communication between AcqKnowledge and Unity projects. For example, data (analog and digital) from the Biopac sensors can be read by Unity in real time and influence the environment, or Unity can control the data collection of AcqKnowledge.

After exploring the capabilities of the Unity Interface utility, the VR Stream, Scenario Stream, and Biopac Stream teams discussed and evaluated that the best approach would be to have Unity add markers in AcqKnowledge based on specific moments of the script, with different symbols and labels, making it easier to analyze the data.

One significant restriction of the Unity Interface to keep in mind is that it is only compatible with the Microsoft Windows (Microsoft Corporation, Redmond, Washington, United States) and Apple macOS platforms (Apple, Inc., Cupertino, California, United States) making it unfeasible to use it with Android (Google LLC, Mountain View, California, United States) (the native platform of the Meta Quest 2 as a standalone device).

Script integration

Working together with the Script Stream team, key points were decided at the beginning of the project that would allow the VR Stream team to develop the scenario while the Script Stream team worked on the actual script. Those key points were: i) the main environments would be an ICU nursing station and patient room, ii) multiple NPCs would talk to the participant, iii) the scenario would follow a dialogue tree, in which the NPCs would ask questions from the participant, and a predefined list of choices would appear, iv) the scenario is a linear story and the dialogue choices are an illusion, meaning that the participants would have no way to avoid doing the action that would go against their morals, and v) there would be survey questions throughout the scenario to gauge the participants’ state.

**Figure 14:**
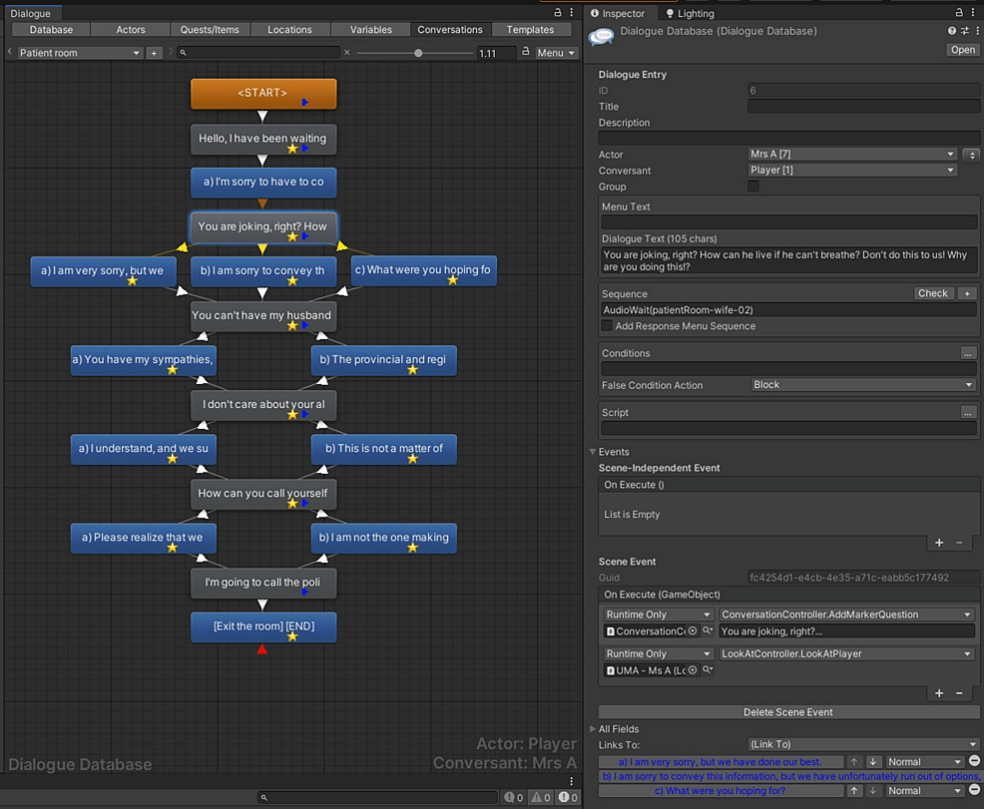
Dialogue tree for the patient room scene, with a question node selected and a sidebar showing its parameters, values, and events.

Based on that information, we implemented the dialogue feature using the Dialogue System for Unity package [21]. The Dialogue System allowed us to quickly implement a tree-based dialogue, with multiple NPCs, scenes, and events triggered when the participant reached specific parts of the dialogue (e.g., adding a marker on AcqKnowledge when the participant reached a question), as shown in Figure 14.

Following is an excerpt of a dialogue between the patient's partner and the participant, listing three choices to choose from.

Patient's partner: You are joking, right? How can he live if he can't breathe? Don't do this to us! Why are you doing this!? Participant: a) I am very sorry, but we have done our best.; b) I am sorry to convey this information, but we have unfortunately run out of options, and our regional algorithm is guiding us.; c) What were you hoping for?

## Discussion

The goal of the VR Stream team on this project was to develop, within a tight deadline (under six months), an interactive and immersive VR scenario that allowed a participant to experience a stressful situation that would have them act against their moral values during the COVID-19 pandemic. To that end, we adopted existing tools, libraries, and packages, modifying them to fit the project's requirements to have a usable version to use in study sessions and collect data from healthcare. As mentioned previously, Nguyen et al. [9] detail the research protocol of the study conducted using the Moral Distress VRS, and Espinola et al. [10] present the final results of the study.

A video of the final version of the Moral Distress VRS (Video 1) shows glimpses of all the components described in this technical report, and we have gained much experience implementing said features.

**Video 1:** Demonstration of the Moral Distress VRS being played by one of the researchers Moral Distress VRS: Moral Distress Virtual Reality Simulator

Most importantly, we would like to highlight the most significant challenges and difficulties faced in implementing each feature. Regarding theenvironment, we recommend conducting test sessions with SMEs and the target audience before starting the actual study, as they can recognize environmental details and elements that are either missing, placed incorrectly, or outright incorrect.

With reference to the characters, although the Daz models provided customizability and the necessary PPE assets, the models were highly detailed. Manually reducing the level of detail is time-consuming, while automatically reducing it can result in artifacts and poor-quality models. Therefore, it is best to plan to create the assets, if possible.

As to the UI and UX, unless specified by the scenario, allow the participant to customize their avatar to help them feel better represented and increase their immersion. In addition, hire experienced voice actors to convey the desired emotions of the NPCs. TTS systems still do not replace voice acting, and inexperienced voice actors may not appropriately portray emotions.

Concerning data collection, running a VR scenario and video recording simultaneously is very taxing on the computer. Extensive testing is necessary to guarantee that the VR scenario performs adequately because frame drops and lag can be disorienting while wearing a VR headset.

Pertaining to the Biopac Stream integration, test early and often to ensure that the connection between Unity and AcqKnowledge is working as expected.

Lastly, regarding the Script Stream integration, getting a draft of the script as soon as possible is vital, as it will guide the development of the environment, characters, assets, and sounds.

If we had to start over, based on the lessons learned, we would focus on having test sessions with SMEs and the target audience earlier in the development cycle to identify possible flaws in the environment and make it more realistic, and reach out to experienced voice actors to record the dialogues.

As future work, we intend to continue developing the Moral Distress VRS platform and perform a literature review, including technical reports, on the development of virtual environments focused on eliciting and monitoring emotions, not limited to stress.

## Conclusions

This technical report presented the development flow of the Moral Distress VRS, developed to research moral distress amongst healthcare workers during the COVID-19 pandemic. It described how different teams worked together and in parallel to achieve the deadline to perform study sessions with participants from the healthcare domain and the decisions that guided the development of each main feature led by the VR Stream team. Furthermore, we presented the challenges faced and possible solutions to overcome them. We expect our experience to guide future teams in quickly achieving high-quality VR-based simulation within a short deadline.
